# How Do Drivers Perceive Risks During Automated Driving Scenarios? An fNIRS Neuroimaging Study

**DOI:** 10.1177/00187208231185705

**Published:** 2023-06-26

**Authors:** Jaume Perello-March, Christopher G. Burns, Roger Woodman, Stewart Birrell, Mark T. Elliott

**Affiliations:** National Transport Design Centre, Centre for Future Transport and Cities, 2706 Coventry University, Coventry, UK; School of Aerospace, Transport and Manufacturing (SATM), 121048 Cranfield University, Cranfield, UK; WMG, 2707 The University of Warwick, Coventry, UK; National Transport Design Centre, Centre for Future Transport and Cities, 2706 Coventry University, Coventry, UK; WMG, 2707 The University of Warwick, Coventry, UK

**Keywords:** aggressive and risky driving, autonomous driving, cognitive neuroscience, human-automation interaction, risk assessment

## Abstract

**Objective:**

Using brain haemodynamic responses to measure perceived risk from traffic complexity during automated driving.

**Background:**

Although well-established during manual driving, the effects of driver risk perception during automated driving remain unknown. The use of fNIRS in this paper for assessing drivers’ states posits it could become a novel method for measuring risk perception.

**Methods:**

Twenty-three volunteers participated in an empirical driving simulator experiment with automated driving capability. Driving conditions involved suburban and urban scenarios with varying levels of traffic complexity, culminating in an unexpected hazardous event. Perceived risk was measured via fNIRS within the prefrontal cortical haemoglobin oxygenation and from self-reports.

**Results:**

Prefrontal cortical haemoglobin oxygenation levels significantly increased, following self-reported perceived risk and traffic complexity, particularly during the hazardous scenario.

**Conclusion:**

This paper has demonstrated that fNIRS is a valuable research tool for measuring variations in perceived risk from traffic complexity during highly automated driving. Even though the responsibility over the driving task is delegated to the automated system and dispositional trust is high, drivers perceive moderate risk when traffic complexity builds up gradually, reflected in a corresponding significant increase in blood oxygenation levels, with both subjective (self-reports) and objective (fNIRS) increasing further during the hazardous scenario.

**Application:**

Little is known regarding the effects of drivers’ risk perception with automated driving. Building upon our experimental findings, future work can use fNIRS to investigate the mental processes for risk assessment and the effects of perceived risk on driving behaviours to promote the safe adoption of automated driving technology.

## INTRODUCTION

Assessing drivers' functional state is essential to ensure the safe adoption of automated driving technology (Alrefaie et al., 2019; Lohani et al., 2019; Perello-March et al., 2021; Wörle et al., 2019). Driver monitoring systems (DMS) will soon be a mandatory safety feature for new production vehicles in Europe (EuropeanCouncil, 2019) and the US (NHTSA, 2022).

Current literature mainly relies on gaze-behaviour and well-established peripheral physiology metrics such as heart rate variability, respiration, blood pressure, and skin conductance (Dong et al., 2011; Lohani et al., 2019; Melnicuk et al., 2021). However, driving and supervising an automated system are primarily cognitive and executive tasks. Much relevant data is overlooked when neurophysiology and neural activity are not measured. Electroencephalography (EEG) is the most well-established neuroimaging technique in driving research (Lohani et al., 2019; Seet et al., 2020; Solís-Marcos et al., 2017, 2018), yet the use of functional near-infrared spectroscopy (fNIRS) in driving research has been increasing over the recent years (Balters et al., 2021). Current fNIRS devices are portable, wearable, lightweight kits, robust to movement artefacts, and allow flexible configurations. In addition, fNIRS provides good spatial resolution but slower temporal resolution than EEG (Balters et al., 2021), making fNIRS an ideal technique for naturalistic research.

Driving research using fNIRS has primarily focused on measuring mental workload and fatigue from manual driving (Foy & Chapman, 2018; Z. Li et al., 2009; Lin et al., 2020; Lohani et al., 2019), but little work has been conducted in the context of driving automation. Only a few studies have used fNIRS to explore constructs such as trust in automation in a driving simulator (Perello-March et al., 2023), where the authors found lower prefrontal activation during hazardous events for participants trusting the automated vehicle compared to those distrusting. Another driving simulator study (Balters et al., 2017) indicated that drivers' prefrontal activity tends to decrease with continuous exposure to automated driving, a phenomenon described as habituation. The habituation to automation increases with higher levels of automation. Relatedly, Sibi et al. (2016) observed a similar decrease in prefrontal activity with automation engaged compared to when driving manually.

These studies suggest that reliable driving automation decrease cortical activity. In short, relegating drivers to a mere monitoring role of a ‘reliable’ system leads to underload and a lack of cognitive capacity to perform an optimal take-over of control – also known as being out-of-the-loop (Merat et al., 2019). This phenomenon could be attributed to situation awareness worsening as the automated system assumes greater control of the driving task (Endsley, 2017). Poor situation awareness and reduced monitoring behaviours due to the shared control between the driver and the automation may put drivers at risk when unexpected take-over requests are issued, especially in conditionally automated driving (SAE Level 3) (SAE International, 2021).

We suspect this poor situation awareness is related to a lower perception of risk resulting from the shared control of the driving task. A lack of risk perception from automated driving is not trivial since it can lead to overtrust and automation misuse (Kundinger et al., 2019; Parasuraman & Riley, 1997), which may compromise safety. Indeed, age-related risky behaviours have previously been explored in traffic psychology research with fNIRS (Foy et al., 2016). The authors found that reduced prefrontal cortex activity during several simulated driving tasks was associated with lower risk perception in young males compared to females and older drivers. The authors attribute these results to a lack of prefrontal maturation in younger male drivers.

Extensive traffic behaviour and psychology work has explored risky driving and driver hazard perception from personality traits (Du et al., 2020; Jonah, 1997). In short, driver hazard perception has been defined as drivers’ situation awareness for potentially dangerous incidents in the traffic environment. That is the ability to detect dangerous traffic situations (Horswill & McKenna, 2004). Effective hazard perception can be considered a central executive task based on a dynamic mental model of the driving environment used for actively searching dangerous situations. A mentally effortful and proactive process that requires working memory and attentional resources (Horswill & McKenna, 2004).

Another related concept that is often confounded with hazard perception is risk perception. It is the subjective evaluation of how well drivers think they – or the driving automation – can handle the situation and apply an appropriate action (Borowsky & Oron-Gilad, 2013). Risk perception has been found to have two major components: (1) the likelihood of a crash and (2) the severity outcomes of a crash (Borowsky & Oron-Gilad, 2013; Riley, 1996). To summarise, hazard perception is the skill to detect hazards in real-time, whilst risk perception is the evaluation of the chances of being involved in a crash in a certain situation. Both have strong ties to situation awareness and trust in automation (Endsley, 1995; Muir, 1994).

In a video-based driving hazard detection and evaluation task experiment, Borowsky & Oron-Gilad (2013) found that drivers would put more weight on the likelihood of a crash in real-time driving. On the other hand, they would pay more attention to the severity of the outcome of a crash when required to evaluate risk in hindsight. The authors argue that under the time pressure of driving, drivers usually focus on preventing the crash (i.e., the likelihood of the crash, the first component of risk perception) rather than thinking of the severity outcomes of the crash (i.e., the second component of risk perception). However, whether these findings are transferable to highly automated driving (HAD, SAE Level 4) remains unclear, where drivers are released from the responsibility and time constraints of real-time driving. To put it in other words, are these components of risk perception equally manifested during HAD? Or instead, drivers remain out of the loop since they are not in control?

Taking the notion that hazard perception is a mentally effortful proactive process involving working memory and attentional resources (Horswill & McKenna, 2004) and that hazard perception is a necessary condition for risk perception to exist (Borowsky & Oron-Gilad, 2013), we considered that cortical neurophysiology could be an adequate research tool to investigate this phenomenon. In particular, cortical haemoglobin oxygenation levels could indicate the underlying central executive cognitive processes involved in real-time risk perception assessments. Previous work using fNIRS has evidenced that cortical haemoglobin oxygenation levels indicate different levels of trust during highly automated driving (Perello-March et al., 2023). Hence, since risk perception is a major factor affecting trust (Lee & Moray, 1992; Muir, 1994; Riley, 1996), cortical haemoglobin concentrations could also be used to measure risk perception in this context.

Borowsky & Oron-Gilad (2013) considered several environmental characteristics, such as the nature of the driving environment – for example, urban or residential, as well as traffic complexity as the combination of environmental features – for example, traffic flow and volume and lane changes among other road users (Teh et al., 2014) to be hazard instigator types, and have been found to increase drivers’ stress levels (Foy & Chapman, 2018; Healey & Picard, 2005; Perello-March et al., 2021). These hazard instigator types can appear in different states of progression – that is, materialised (require an evasive response), hidden unmaterialised (obscured by other road objects but not require a response), and potential unmaterialised (visible but not require response). Thus, we have conducted a high-fidelity driving simulator study with two types of hazard instigators:

(1) Potential unmaterialised hazards across suburban and urban driving conditions with moderate levels of traffic complexity slowly building up.
(2) A materialised hazard in a quickly escalating driving scenario requiring an evasive manoeuvre.

We expect cortical prefrontal oxygenation levels to increase if drivers actively search for potential hazards and evaluate the likelihood and severity of the outcomes of a potential crash. On the contrary, low perception of risk should result in a decrement in prefrontal cortical activity. Based on the findings described in the literature review, we hypothesise:

Hypothesis 1: Prefrontal haemoglobin oxygenation levels during suburban and urban driving conditions with moderate levels of traffic complexity and potential un-materialised hazards will not differ from baseline resting and a recovery period due to a lack of situation awareness and perceived risk.

Hypothesis 2: The materialised hazard in a quickly escalating driving scenario requiring an evasive manoeuvre will produce variations in prefrontal haemoglobin oxygenation compared to the baseline resting and recovery period due to perceived risk.

## METHOD

### Participants

A convenience sample of twenty-three volunteers was recruited to participate in this experiment. Three participants were excluded from the analysis as they dropped out from the experiment due to motion sickness, with the data of 20 participants analysed (10 female, M_age_ = 24.60, SD = 3.91). All had held a UK-EU driving license for an average of 5.30 years (SD = 4.18) and an average driving experience of 6780 miles/year (SD = 6140.08). Participants were recruited from the University of Warwick (UK), including undergraduate and postgraduate students and professionals. Recruitment and data collection procedures received approval from the University of Warwick’s Biomedical and Scientific Research Ethics Committee. Participants were free to withdraw at any point and did not receive compensation.

### Driving Simulator

**Figure 1.**
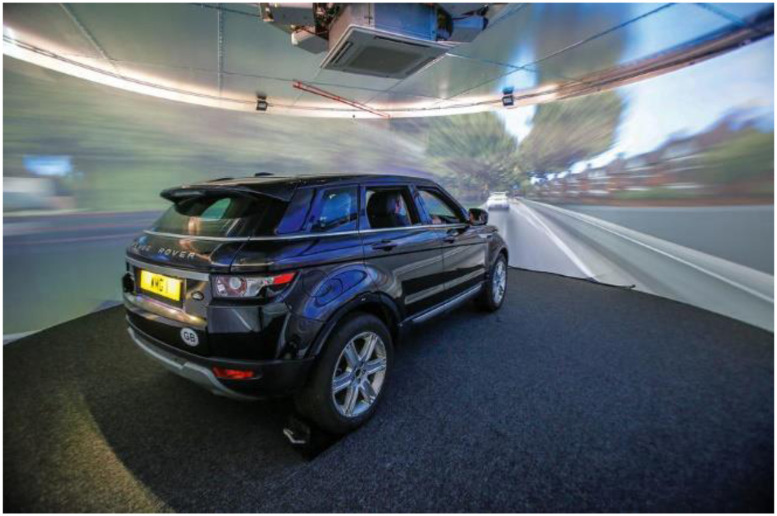
Snapshot of the driving simulator and the virtual environment projected on a curved screen.

The experiment took place in the driver-in-the-loop 3xD driving simulator at WMG, the University of Warwick. The 3xD (Figure 1) is a fixed-base high-fidelity driving simulator with a whole-body Range Rover Evoque and 8 projectors generating a 360° image projected into a cylindrical screen 8 metres in diameter and 3 meters in height. The simulated driving automation is capable of lateral and longitudinal control, adapting to speed limits, queuing leading vehicles, maintaining safe distances, emergency braking, and overtaking slower/stopped vehicles for predefined use cases. The road environment sound and motion vibration are played stereo via 2 × 20 W speakers.

### Functional Near-Infrared Spectroscopy

**Figure 2.**
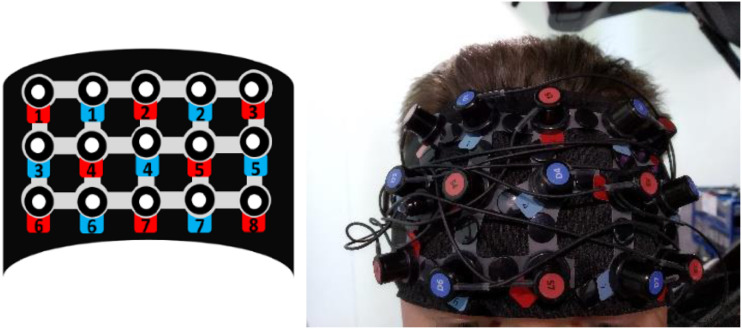
Channels montage and representation of the whole setup device – in our case, the hardware was placed behind the passenger seat.

Neurophysiological data was obtained from the prefrontal cortex with a NIRSport CW-NIRS device (NIRx Medical Technologies LLC, USA) (Figure 2), using NIRStar acquisition software (CA, USA; version 15.0). NIRSport is a noninvasive wearable device consisting of eight sources and seven detectors sampling at a frequency of 7.8125 Hz. The sources simultaneously emit infrared signals of two distinct wavelengths, 760 nm and 850 nm, allowing quantification of oxygenated haemoglobin (HbO), deoxygenated haemoglobin (HbR), and total haemoglobin (HbT = HbO + HbR). Both chromophores can be differentiated when light attenuation is measured at two or more wavelengths due to their differential absorption spectra in the near-infrared spectrum (600–950 nm).

Plastic spacers located at a distance of 3 cm between each source and detector pair constitute a recording channel, thus resulting in 22 recording channels. Channels were mounted within the Brodmann areas coordinate space for consistency across head size variation using the fNIRS Optodes’ Location Decider (fOLD), which is a toolbox for probe arrangement guided by brain regions of interest. The toolbox automatically decides optodes positions based on 10–10 and 10–5 systems according to a set of brain regions of interest (Zimeo Morais et al., 2018). These coordinates allow subsets of fNIRS channels to directly measure particular regions of interest (ROIs) (Table 1)

**TABLE 1.** List of Channels and Regions of Interest

Source	Detector	Channel	Brodmann Area
1	1	1	8 right
1	3	2	-
2	1	3	8 right
2	2	4	8 left
2	4	5	8 left
3	2	6	8 left
3	5	7	44 left
4	1	8	8 right
4	3	9	9 right/46 right
4	4	10	9 left/9 right
4	6	11	9 right
5	2	12	-
5	4	13	9 left
5	5	14	44 left/45 left
5	7	15	10 left
6	3	16	46 right
6	6	17	10 right/46 right
7	4	18	9 left
7	6	19	10 right
7	7	20	10 left
8	5	21	45 left
8	7	22	45 left/46 left

### Questionnaires

Subjective measures included a bespoke risk perception questionnaire comprising two items which were asked after completing the trial:

(1) Did you feel any sensation of risk or threat from the whole scenario?
(2) Did you feel any sensation of risk or threat from the traffic accident at the end?

These were rated on a Likert scale ranging from 1 (not at all) to 7 (extremely).

Current validated measures for hazard perception are image/video-based tests in which participants are asked to detect promptly potential hazards from several road scenarios (Joanne et al., 2010; Malone & Brünken, 2016). Hazard perception performance is measured by latency (response time) and accuracy (success/failure to detect). However, to the authors’ knowledge, no existing validated self-reported tools for risk perception assessment in the driving context exist.

Li et al. (2019) used the scale from Rajaonah et al. (2008) to assess risk perception associated with trusting in automation. Even though both studies reported significant effects on risk perception, we did not use this scale because it needs to be validated and measures perceived situational and relational risk. In our experiment, we were interested in comparing perceived situational risk with the hazardous event during the automated driving scenario. Thus, this would have implied reporting perceived risk at the end of each condition, which we considered was contraindicated due to our continuous driving experimental design and as the hazardous event occurred immediately after the automated driving conditions. Stopping the scenario immediately before the Driving Hazard event could have affected the realism of the scenario and any neurophysiological reactions.

In addition, the Trust in Automated Systems Scale (Jian et al., 2000) was included to evaluate the perceived risk’s impact in building trust in automation. The scale was rated before and immediately after the trial was completed.

### Experimental Procedure

**Figure 3.**
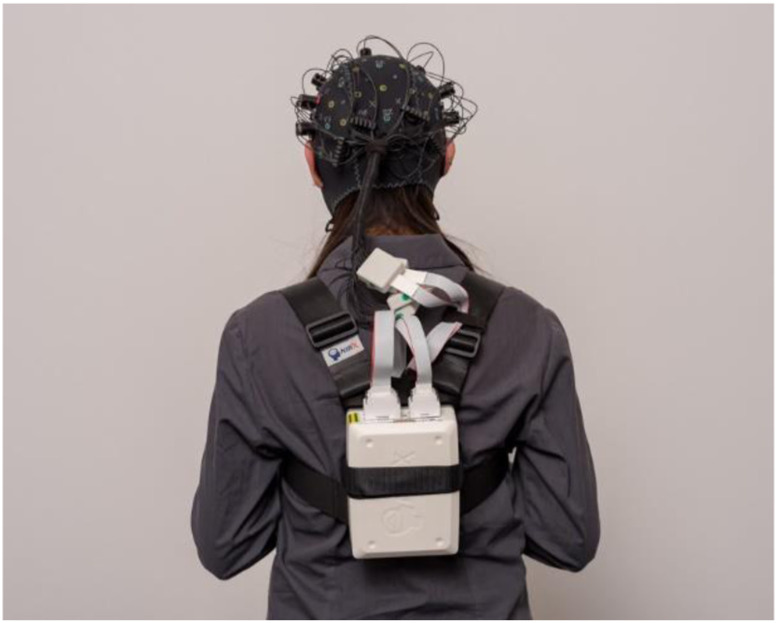
fNIRS equipment. In our experiment, we attached the backpack to the back of the driver’s seat. Source: nirx.net.

Upon arrival, participants were guided into the simulator control room, where they were briefed on lab safety procedures and filled in the consent form and demographic inventories. Participants were then guided inside the driving simulator. They were informed that the experiment would start by recording their resting physiological state baseline for 4 minutes, and after that, the driving scenario would begin. They were asked to remain seated in the driver’s seat, not move excessively, to breathe normally, and stay relaxed during the baseline recording. Participants were advised that the experimenter would inform them of the start and end of the baseline recording. The driving simulator lights were switched off, the room was silent, and driving scenarios were not projected on the screen. The fNIRS data recording hardware was placed in a backpack behind the driver’s seat (Figure 3). The fNIRS headset was attached when the participant was seated and calibrated to start the baseline recording. After recording the baseline, the automated driving scenarios lasted approximately 5 minutes. The total duration of the experiment was 11 minutes and 30 seconds.

Participants were instructed to sit in the driver’s seat but were not explicitly asked to monitor the environment. Instead, they were asked not to engage in the driving task. The rationale for doing this was that they were about to test a highly automated vehicle that they did not need to drive manually, nor would they be requested to take over. Participants were not free to perform other tasks, as this could disrupt their situation awareness or affect their neurophysiology.

**Figure 4.**
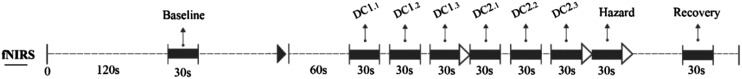
Experimental conditions in order of occurrence. Shaded boxes indicate data analysed.

The automated driving scenarios were split into two segments. An initial two-minute suburban driving scenario labelled Driving Condition 1 was split into 30-second blocks for fNIRS analysis, with interblock intervals of 15 seconds (Figure 4). These segments were thus referred to as DC1.1, DC1.2, and DC1.3. Driving scenarios started with the ego vehicle stopped at a red traffic light at a five-lane roundabout, which carries traffic to and from the highway to the suburbs and the city centre. This initial portion of the scenario lasted 60 seconds and served as a familiarisation so participants could adjust to the driving simulation. The ego vehicle took the third roundabout exit leading to a straight dual carriageway, separated by a central reservation. Speed was limited from 30 to 50 mph. Surrounding traffic levels were low (<5 road users per minute), and weather conditions were cloudy. Approximately 1 minute later, the ego vehicle entered the suburbs. This layout consisted of two lanes passing through residential areas at a maximum of 30 mph, including several left and right turns and give-way exits. Oncoming traffic increased to medium levels (<20 road users per minute), including pedestrians, cyclists, and parked cars, on the roadside and driveways.

The simulation continued with a two-minute city centre scenario, denoted as Driving Condition 2, that is, DC2.1, DC2.2, and DC2.3, as these were also split into 30-second blocks with interblock intervals of 15 seconds (Figure 3). In this scenario, the layout changed to a ‘high street’ area surrounded by commercial buildings, signs, and billboards. It also implied higher levels of moving pedestrians and vehicles, including vans and buses, stopped on the roadside – which the vehicle had to overtake – and T-junctions with traffic approaching from both directions (between 20 and 40 road users per minute). The speed limit was 30 mph, and the weather shifted to heavy rain, degrading the visual range.

Finally, the Driving Hazard event occurred when leaving the city centre to enter the suburbs again, on the approach of a T-junction, in a residential area from a straight two-way lane. This event was the sudden appearance of a heavy single-cabin semitrailer truck, which accelerated into the scene at high speed (60 mph) from the left side of the T-junction ahead, moving sideways and heading directly towards the ego vehicle. The ego vehicle performed a sudden evasive manoeuvre to avoid the trailer, steered to the right side and collided with a garden fence. This sequence (i.e., from leaving DC2 to the end of the crash) occurred over 30 seconds. After the hazardous event, participants remained in the vehicle with the scenario displayed on-screen for 2 minutes to record a postevent recovery. Afterwards, the experimenter entered the simulator and accompanied them back into the control room to fill in the risk perception and the trust in automation scale.

### Data Preprocessing

Raw fNIRS data were preprocessed using HomER 3 (Huppert et al., 2009) scripts running on MATLAB R2019a (Mathworks Inc.) according to the current recommendations for preprocessing fNIRS data (Pinti et al., 2019) (Table 2). For current best practices and publication guidelines see Yücel et al., 2021. Corrected optical density data were then converted to HbO, HbR, and HbT concentrations using the modified Beer–Lambert law. Once optical density concentrations were calculated, data was block-averaged and exported as haemodynamic response function (HRF) means.

**TABLE 2.** Data Preprocessing

Step	Description	Function	Input Values
1	Remove channels in which the signal was too weak, too strong, or their standard deviation was too great	hmrRPruneChannels	dRange = 1 × 10^4^ 1e + 07 SNRthresh = 5 SDrange = 0.0 to 45.0
2	Transforms fNIRS raw data into optical density	hmrRIntensity2OD	None
3	Identifies and corrects motion artefacts	hmrRMotionCorrectPCArecurse	tMotion = 0.5 tMask = 1 STDthresh = 20 AMPthresh = 5 nSV = 0.97 maxIter = 5
4	Eliminates noise from physiological activity, low-frequency signal drifts or machine noise.	hmrRBandPassFilt	hpf = 0.01 lpf = 0.5
5	Converts optical density to concentrations	hmrROD2concNew	ppf = 1 (760 nm) 1 (850 nm)
6	Calculates the block average of the given conditions	hmrR_BlockAvg	None

### Data Analysis

Block-averaged HbO and HbR values from HomER 3 were exported in excel files containing HRF means for each channel, condition, and participant. The underlying ROIs were determined using the NIRS Brain AnalyzIR toolbox (Santosa et al., 2018) to calculate the corresponding anatomical labels for each position. The toolbox creates a variable that lists the channels and BAs covered by the probe and the relative ‘weights’ for each channel and BA. The weights for each BA add up to 1. The channel with the most sensitivity to a BA has the highest weight for that area. The relative weight is a helpful metric, but it does not give the complete picture, so we also extracted a ‘depth’ value for each channel and BA. Depth values represent the distance on average between the channel and the BA – that is, the further the distance, the lower the likelihood that the channel captures that BA. Therefore, we selected up to three channels accounting for at least a combined relative weight of 0.80 (i.e., covering at least 80% of a particular ROI) and for the lowest combined depth value (i.e., the smallest combined distance on average).

The rationale for not averaging all channels with a relative weight greater than 0 for a given BA is that some values are too low. If too many channels are averaged together, the response will be negated. Following Wiggins et al. (2016), we established averaging only up to 3 channels together. The most sensitive channels of each ROI were grouped. This led to 10 ROIs: Bilateral BAs 08, 09, 10, and 46, and left BA44 and 45. Having grouped the relevant channels into ROIs, values were averaged within each ROI for each experimental condition, resulting in seven means (one per experimental condition) per participant for each ROI and each chromophore (Table 1). These concentration values were then standardised to enable interindividual and intraindividual comparisons using Z-scores (M = 0; SD = 1). Each single mean concentration value was then transformed into Z-scores against the mean group baseline value and its standard deviation (i.e., Z = (X – baseline mean)/baseline SD) (Table 3). Data standardisation is a common procedure among fNIRS studies to allow for interindividual comparisons in parametrical statistical analysis using block-averaged values (Durantin et al., 2014; Leon-Dominguez et al., 2014; Lin et al., 2020; Minematsu et al., 2018; Roche-Labarbe et al., 2008; Tanida et al., 2004; Verdière et al., 2018).

**TABLE 3.** Data Analysis

Step	Description	Function	Criteria
1	Determining underlying ROIs for each channel	nirs.util.converlabels2roi	
2	Determining sensitivity for each channel using relative weights and depth values	nirs.util.depthmap	
3	Determining most sensitive channels combined for each ROI		Up to three channels with the highest relative weight and lowest depth values
4	Averaging most sensitive channels for each ROI and for each experimental condition	Mean	
5	Standardising individual mean HRF concentrations	Z = (X – baseline mean)/baseline SD	
5	Test for normality assumption	Shapiro–Wilk	*p* > 0.05
6	Test for sphericity assumption	Mauchly	*p* > 0.05
7	Test for main statistical effects and interactions using mixed repeated-measures ANOVA	Analysis of variance	*p* < 0.05
8	Follow-up pair-wise comparisons	Bonferroni correction	*p* < 0.05

The analysis of variance (ANOVA) is a common technique to determine localised brain activation based on changes in simultaneous HbO and HbR concentrations in repeated measures and block designs (Balters et al., 2021; Tak & Ye, 2014). Although it is common in the literature to report only HbO, HbR or HbT, the haemodynamic response is bi-dimensional. HbO and HbR usually correlate negatively during brain stimulation because increased blood flow produces an increase in oxygenated haemoglobin and a decrease in deoxygenated haemoglobin (Fallgatter & Strik, 1998; Mehagnoul-Schipper et al., 2002; Schroeter et al., 2002; Taga et al., 2003). Nonetheless, since these features may not necessarily be reciprocal, several authors have argued that interpretations based exclusively on one chromophore would be incomplete and advocate reporting both features in tandem (Liu et al., 2016; Obrig, 2010; Quaresima et al., 2012). Therefore, following these recommendations, repeated measures ANOVAs were conducted to determine changes in haemoglobin concentrations on each chromophore separately.

HbO, HbR, and HbT mean HRF concentrations grouped in ROIs were imported and analysed with IBM SPSS Statistics 26 software. The significance level was set at α < 0.05. The Shapiro–Wilk’s test (p ≥ 0.05) was used to assess normality assumption violations, and Mauchly’s test was used to assess the assumption of sphericity. Thus, repeated-measures ANOVAs were conducted for each ROI individually (i.e., BAs 8, 9, 10, and 46 bilateral, plus BAs 44 and 45 on the left hemisphere). Main effects and interactions were followed-up by Bonferroni-corrected pair-wise comparisons.

## RESULTS

This block design experiment investigated whether variations in perceived risk from either slowly evolving or rapidly evolving driving conditions would produce observable changes in neurophysiology. Two participants were excluded from the analysis due to significant noise in raw data (N = 18).

### Hypothesis 1 – Perceived Risk From Slowly Evolving Conditions

This hypothesis tested whether slow changes across Driving Conditions would produce oxygenation concentration variations within participants. We ran a repeated-measures ANOVA with 8 levels (BL, DC1.1, DC1.2, DC1.3, DC2.1, DC2.2, DC2.3, and Recovery; see Figure 4) on HbO, HbR and HbT, but HbR did not report any statistically significant effects.

**Figure 5.**
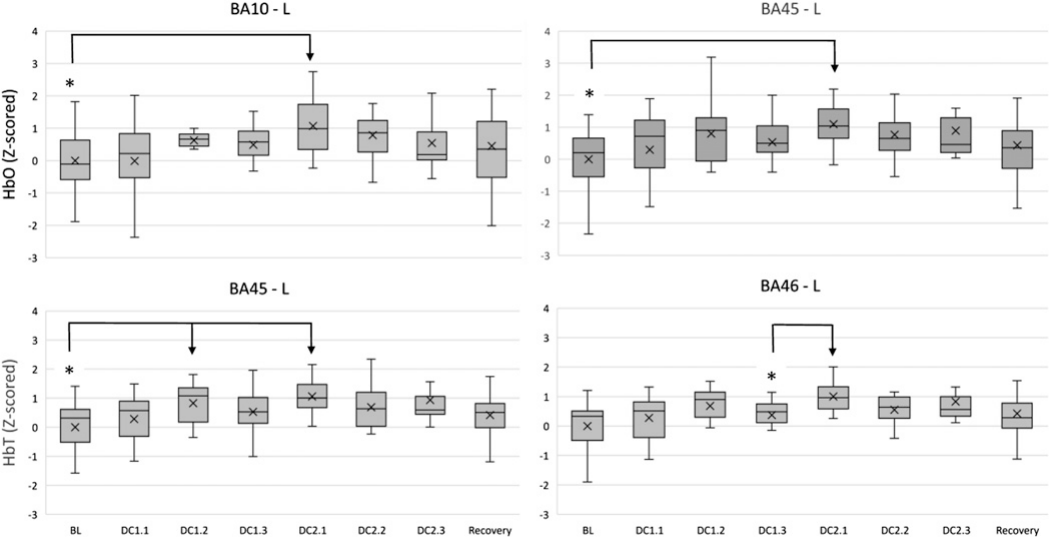
HbO levels in BA10-left and BA45-left (top), and HbT levels in BA45-left, BA46-left (bottom); across moderate risk driving conditions. Asterisks (*) indicate main effects for condition. Mean is indicated by (x). Error bars indicate standard error.

A main effect for Driving Conditions was observed in the left orbitofrontal cortex (BA10-L) for HbO (F (7, 119) = 2.330, *p* = 0.029, η^2^p = 0.121, Figure 5). Post hoc tests indicated an increase in oxygenation from BL (0.000 ± 1.000) to DC2.1 (1.067 ± 0.880, *p* = 0.020).

BA45, located in the left ventrolateral cortex, reported a main effect for Driving Conditions on HbO (F (7, 119) = 2.197, *p* = 0.039, η^2^p = 0.114, Figure 5). Post hoc tests indicated an increase in oxygenation from BL (0.000 ± 1.000) to DC2.1 (1.093 ± 0.660, *p* = 0.011). Furthermore, HbT levels also varied within Driving Conditions (F (7, 119) = 2.827, *p* = 0.032, η^2^p = 0.143, Figure 5), with post hoc comparisons revealing an increase from BL (0.000 ± 1.000) to DC1.2 (0.829 ± 0.671, *p* = 0.039) and DC2.1 (1.061 ± 0.554, p = 0.008).

BA46-L, located in the left dorsolateral cortex, reported a main effect for Driving Conditions on HbT (F (7, 119) = 2.902, *p* = 0.037, η^2^p = 0.146, Figure 5), with post hoc comparisons revealing an increase from DC1.3 (0.372 ± 0.592) to DC2.1 (1.006 ± 0.462, *p* = 0.049).

**Figure 6.**
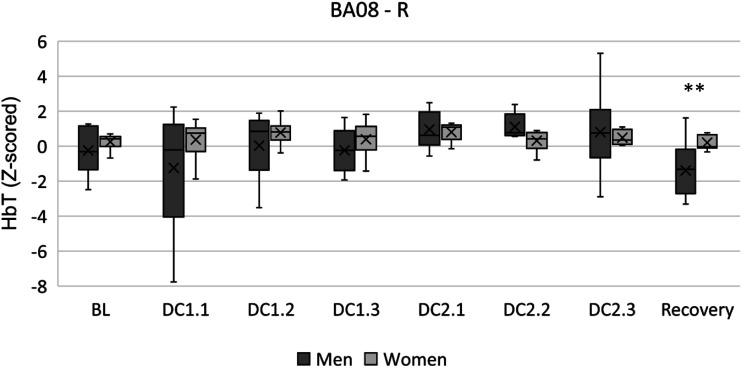
HbT levels in BA08 – right between men and women across moderate traffic complexity driving conditions. Double asterisks (**) indicate main effects between subjects. Mean is indicated by (x). Error bars indicate standard error.

A further exploration with participant sex as independent variable reported a main effect on the right anterior premotor cortex (BA08-R) for HbT (F (1, 16) = 4.541, *p* = 0.049, η^2^p = 0.221). Pair-wise comparisons revealed that Women registered significantly higher levels of HbT (0.199 ± 0.407, *p* = 0.011) than Men (−1.390 ± 1.609) during the postevent recovery period Figure 6.

### Hypothesis 2 – Perceived Risk From Rapidly Evolving Conditions

The second hypothesis investigated whether the rapidly evolving Driving Hazard event would produce observable effects in oxygenation concentrations within participants. To analyse the effect of the rapidly evolving Driving Hazard event (H2), we ran a repeated measures ANOVA with three levels (BL, Driving Hazard, and Recovery).

Strong evidence in favour of H2 was found across HbO and HbT; however, HbR did not report any statistically significant effect. A main effect for Driving Hazard on HbO (F (2, 34) = 4.418, p = 0.020, η^2^p = 0.206) and HbT (F (2, 34) = 3.470, p = 0.043, η^2^p = 0.170) was found on BA09-R, but these effects diminished in post hoc tests.

**Figure 7.**
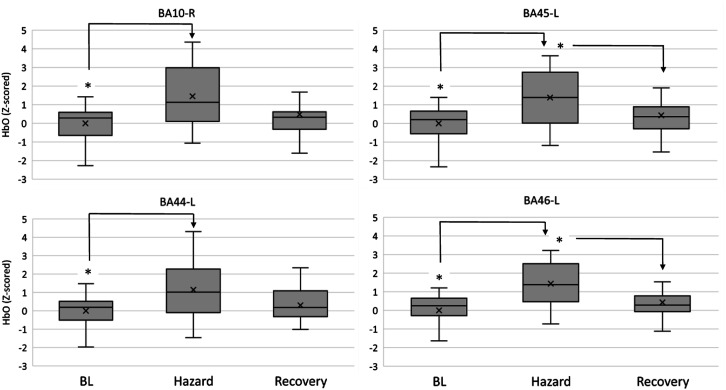
HbO levels in BA10-right and BA45-left (top), and in BA44-left, BA46-left (bottom); between the driving hazard condition and resting periods. Asterisks (*) indicate main effects for condition. Mean is indicated by (x). Error bars indicate standard error.

Lateralised orbitofrontal activation was observed, with BA10-R reporting a main effect of Driving Hazard on HbO (F (2, 34) = 5.846, *p* = 0.007, η^2^p = 0.256, Figure 7) and post hoc tests revealing an increase from BL (0.000 ± 1.000) to Hazard (1.451 ± 1.661, *p* = 0.021). HbT also showed this effect (F (2, 34) = 4.118, *p* = 0.025, η^2^p = 0.195), although diminishing with post hoc tests.

**Figure 8.**
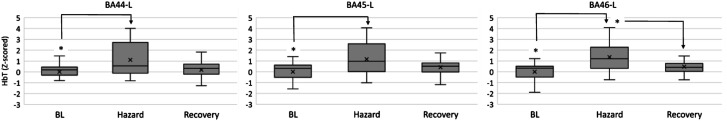
HbT levels in BA44-left, BA45-left, and BA46-left between the driving hazard condition and resting periods. Asterisks (*) indicate main effects for condition. Mean is indicated by (x). Error bars indicate standard error.

BA44 reported a main effect of Driving Hazard on HbO (F (2, 34) = 6.968, *p* = 0.007, η^2^p = 0.291, Figure 7) with post hoc tests revealing an increase from BL (0.000 ± 1.000) to Hazard (1.145 ± 1.530, *p* = 0.019). HbT seconded this effect (F (2, 34) = 6.691, *p* = 0.011, η^2^p = 0.282, Figure 8), with post hoc tests revealing a similar increase from BL (0.000 ± 1.000) to Hazard (1.111 ± 1.579, *p* = 0.030).

BA45 reported a main effect of Driving Hazard on HbO (F (2, 34) = 10.950, *p* < 0.001, η^2^p = 0.392, Figure 7) with post hoc tests revealing an increase from BL (0.000 ± 1.000) to Hazard (1.387 ± 1.455, *p* = 0.002), and followed by a decrease from Hazard to Recovery (0.436 ± 1.060, *p* = 0.038). HbT seconded this effect (F (2, 34) = 7.559, *p* = 0.002, η^2^p = 0.308, Figure 8), with post hoc tests revealing a similar increase from BL (0.000 ± 1.000) to Hazard (1.162 ± 1.503, *p* = 0.013).

The dorsolateral prefrontal cortex showed bilateral activity during the Driving Hazard event. BA46-L reported a main effect of Driving Hazard on HbO (F (2, 34) = 11.743, *p* < 0.001, η^2^p = 0.409, Figure 7) with post hoc tests revealing an increase from BL (0.000 ± 1.000) to Hazard (1.434 ± 1.227, *p* = 0.002), and followed by a decrease from Hazard to Recovery (0.427 ± 0.916, *p* = 0.017). HbT seconded this effect (F (2, 34) = 9.008, *p* = 0.001, η^2^p = 0.346, Figure 8), with post hoc tests revealing a similar increase from BL (0.000 ± 1.000) to Hazard (1.356 ± 1.386, p = 0.009), and a posterior decrease from Hazard to Recovery (0.467 ± 0.898, p = 0.046). BA46-R reported a main effect of Driving Hazard on HbO (F (2, 34) = 5.760, p = 0.017, η^2^p = 0.253), although fading away with post hoc comparisons.

**Figure 9.**
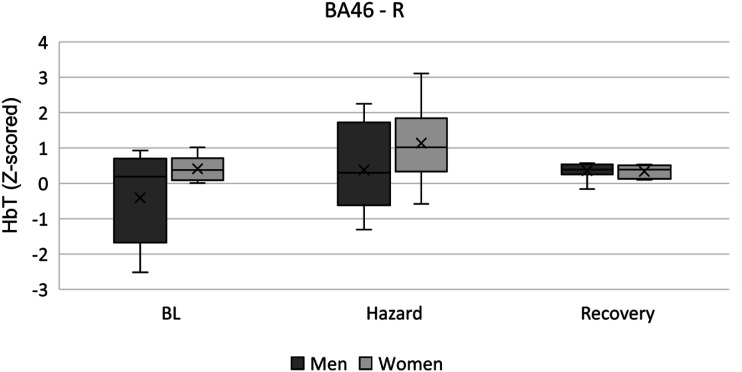
HbT levels in BA46 - right between men and women during the driving hazard condition and resting periods. Mean is indicated by (x). Error bars indicate standard error.

Finally, a further exploration with sex as independent variable reported a main effect for HbT in the right dorsolateral prefrontal cortex (BA46-R) (F (1, 16) = 5.590, p = 0.031, η^2^p = 0.259 Figure 9); however, this effect diminished after pair-wise comparisons. Nonetheless, it is worth noting that descriptive data indicate higher levels of HbT on women (1.142 ± 1.088) than men (0.380 ± 1.274) during the event.

### Psychometric Results

Finally, a Wilcoxon signed-rank test reported a main effect for self-reported risk perception (Z = 194.5, p = 0.001), with perceived risk during the Driving Hazard event (Mdn = 5.50, IQR = 3) being significantly higher than during Driving Conditions (Mdn = 3.00, IQR = 3).

Results from the TASS scale revealed a main effect for trust (F (1, 18) = 5.975, p = 0.025, η^2^p = 0.249), indicating a significant increase in trust ratings after the trial (4.721 ± 1.207, p = 0.025) compared to before the trial (4.279 ± 0.893).

## DISCUSSION

### Hypothesis 1

This empirical research investigates the effect of traffic complexity on drivers’ perceived risk measured during highly automated driving through prefrontal haemoglobin oxygenation concentrations.

The first hypothesis investigated whether suburban and urban driving conditions with moderate levels of traffic complexity and potential unmaterialised hazards would produce variations in brain oxygenation within participants. Moderate risk during these was assumed as the median of self-reported perceived risk across Driving Conditions was 3 out of a maximum of 7.

The null hypothesis was rejected since oxygenated and total haemoglobin (HbO and HbT) concentrations increased significantly in the orbitofrontal (BA10), ventrolateral (BA45) and left dorsolateral (BA46) areas would be consistent with supervising the automated vehicle’s performance when transitioning from the suburbs (DC1.2 and DC1.3) to the city centre (DC2.1) (Figure 5).

The orbitofrontal region has been found to activate when operators are judging the trustworthiness of automated systems (Palmer et al., 2020; Perello-March et al., 2023) and as an indicator of the willingness to intentional engagement (Dimoka, 2010), thus perhaps indicating an intention of taking-over manual control during these scenarios.

Increased oxygenation in the ventrolateral cortex has been associated with distrust (Hubert et al., 2018; Palmer et al., 2020), state-level suspicion (Hirshfield et al., 2014), and frustration during automated driving (Damm et al., 2019). In addition, this area is related to intense negative emotions of distrust, fear and negative consequences (Dimoka, 2010; Hubert et al., 2018).

The left dorsolateral region reported HbT variations from the suburbs (DC1.3) to the city centre (DC2.1). This area is implicated in decision-making from perceptual inputs and has been associated with trust calibration (Drnec & Metcalfe, 2016; Hubert et al., 2018; Palmer et al., 2020). Perhaps participants perceived the change in traffic complexity between driving conditions 1 and 2 (i.e., from suburbs to the city centre) and evaluated the automated vehicle performance and trustworthiness when entering such a new driving context. However, self-reported data showed that perceived risk across driving conditions remained moderate.

Finally, an exploration of sex differences indicated that women registered significantly higher levels of HbT in the right anterior premotor cortex (BA08) during the recovery phase. This area has been found to activate when participants experience uncertainty (Volz et al., 2005). Moreover, these results would align with previous work finding that females show increased lateralised right cortical activity and are more alert during complex traffic conditions (Foy et al., 2016).

Findings from H1 suggest drivers actively monitored the road for potential hazards during moderate traffic complexity conditions. Drivers showed increased dorsolateral, ventrolateral, and orbitofrontal cortical oxygenation that could be attributed to the awareness of un-materialised potential road hazards. Considering that they were not actively instructed to remain responsible for the driving task, we suggest the increased activation in these cortical areas could be attributed to suspicion towards the vehicle’s trustworthiness and the evaluation of the likelihood of a crash – that is, the first component of risk perception – and therefore, participants could be actively calibrating their trust in the automated vehicle.

### Hypothesis 2

The second hypothesis predicted that the materialised hazard in a quickly escalating driving scenario requiring an evasive manoeuvre would significantly increase haemoglobin oxygenation compared to baseline resting and postevent recovery resting. Substantial evidence favouring hypothesis 2 was found as participants reported significantly greater risk during the Driving Hazard (Mdn = 5.5/7) than during the Driving Conditions (Mdn = 3/7), supported by robust increases throughout Hbo and HbT.

As in hypothesis 1, bilateral orbitofrontal (i.e., BA10) activation during the Driving Hazard could be attributed to actively judging the automated vehicle trustworthiness derived from an increased risk perception (Dimoka, 2010; Palmer et al., 2020). Consistent activation of this cortical area due to factors related to automated driving performance – for example, traffic complexity and driving conditions – also in Perello-March et al. (2023) reinforce the notion that the orbitofrontal cortex plays a crucial role in judging the trustworthiness of automated vehicles during uncertain situations – that is, trust calibration. This cortical region likely acts as a ‘comparator’ for perceptual information and vehicle reliability, from which situational trust is derived.

Further evidence supporting this statement was found in the left ventrolateral prefrontal cortex (BA44/BA45). Increments in both HbO and HbT from baseline to Hazard scenarios are indicative of attention allocation during visual search (Anderson et al., 2007), increment in distrust (Hirshfield et al., 2014; Hubert et al., 2018; Palmer et al., 2020), and experiencing strong unpleasant emotions (Hirshfield et al., 2014; Hoshi et al., 2011; Hubert et al., 2018). Likely, our participants actively sought visual cues to analyse, understand and predict the potential consequences of the sudden Driving Hazard event, which might be the anticipation of unpleasant emotions such as momentary fear and distrust.

Notably, the insular cortex in the inferior frontal gyrus – near BA45 – is often called the centre for risk perception. It has been associated with decisions with strong negative emotional components (Dimoka, 2010; Hubert et al., 2018), possibly an evolutionary trace to prevent negative interactions and their consequences (Kahneman & Tversky, 1983, 2019; Rangel et al., 2008). Moreover, the insula seems strongly related to a cognition-based mechanism for risk assessment of contextual information and their appraisal (Hubert et al., 2018; Singer et al., 2009). BA44/45 are close to the insular cortex.

Bilateral activation was also observed in the dorsolateral prefrontal cortex (i.e., BA46-L, BA46-R, and BA09-R), reporting HbO and HbT increases from baseline to Hazard, followed by decreases from Hazard to Recovery phases in both measures. Dorsolateral prefrontal cortex activity is often attributed to deliberate decision-making and reflective processes related to trust (Dimoka, 2010; Drnec et al., 2016; Hubert et al., 2018) and supporting the orbitofrontal cortex in comparing uncertain perceptual – for example, visuospatial – information (Bruno et al., 2018). Therefore, as in the orbitofrontal cortex, higher activation of the dorsolateral prefrontal cortex would be related to assessing the trustworthiness of the automated vehicle when relevant contextual changes occur (Hubert et al., 2018; Perello-March et al., 2023). Besides, the dorsolateral prefrontal cortex has been attributed to play an essential role in emotional regulation and self-control (Hirshfield et al., 2014; Hubert et al., 2018). Activation of this area after experiencing a sudden strong negative emotion – fright, startle, fear – derived from the Hazard event would indicate emotional regulation. Supporting this claim, we observed a significant decrease in HbO and HbT during the recovery phase.

Finally, the evaluation of sex-based differences in cortical oxygenation showed increased HbT levels in the right dorsolateral prefrontal cortex (BA46) in women during the recovery phase. This finding aligns with those in H1, indicating a lateralised right dorsolateral and premotor cortex activation in women not present in men. As mentioned, prefrontal hemispheric lateralisation for women during hazardous driving conditions was also observed in an fNIRS study conducted by Foy et al. (2016).

Results from H2 indicate that drivers perceived the hazard but also evaluated the likelihood and the severity of the outcome of the crash. This was inferred since drivers reported higher risk and we observed the activation of areas related to emotion regulation and the anticipation of negative consequences derived from the severity of the crash. Since drivers were freed from the real-time driving task, they could allocate cognitive resources to evaluate how well the driving automation could handle the situation and apply an appropriate action. Hence, we suggest that contrary to manual driving, when drivers evaluate the ability of the highly automated driving to handle a materialised hazardous situation, both components of risk perception – the likelihood and the severity of the crash – are present.

These results build on previous neurophysiology and traffic psychology work using fNIRS relating risk-taking behaviours while driving and age, with young males showing a lack of prefrontal maturation that may explain the increased crash risk seen in this population (Foy et al., 2016). Measuring drivers’ prefrontal activation with automated driving could be used to investigate risk-taking behaviours in future work. Whereas risky driving behaviours have been extensively investigated in previous traffic behaviour psychology (Jonah, 1997), little work has investigated risk-taking behaviours and driver hazard perception under automated driving. Therefore, our findings are of particular novelty and relevance to shed light on this technology’s new human factors–related challenges and comprehend how automated driving users perceive risks.

In addition, we observed an increase in the self-reported trust after the trial. Whilst this finding may seem contradictory to the fact that perceived risk was higher towards the end of the trial. One could expect trust to be low in such a context. It can be argued that the TASS scale measures propensity to trust (i.e., dispositional trust) rather than situational trust (Adams et al., 2003; Holthausen et al., 2020). Hence, such increment in dispositional-learned trust could be attributed to familiarisation due to mere exposure to the automated driving system and the fact that the hazardous event was negotiated successfully. Similar trust increments after a short exposure to automated driving have also been reported in previous studies (Dixon et al., 2019; Gold et al., 2015; Kraus et al., 2020; Kundinger et al., 2019; Large et al., 2019; Lee et al., 2021). The findings evidenced in this paper suggest that situational perceived risk does not necessarily affect dispositional and learned trust, which other factors would modulate (for a review of trust layers see Hoff & Bashir, 2015). Whereas a higher situational risk perception is expected to correlate negatively with lower situational trust (Li et al., 2019), this may not necessarily apply to dispositional and learned trust.

Our results demonstrate that fNIRS is valid for measuring variations in perceived risk from traffic complexity during highly automated driving, particularly HbO and HbT measures. The lack of significant effects for HbR data is not unusual since HbR is known to be a less robust parameter (Balters et al., 2021). Increased oxygenation in prefrontal areas would indicate active monitoring of the driving performance reliability in complex traffic scenarios. Even though our drivers were not responsible for the driving task and dispositional trust was high, they showed variations in perceived risk as traffic complexity built up gradually, especially during the driving hazard. This suggests our drivers were ‘in the loop’ and would have been able to resume manual control if required.

### Limitations and Future Work

Using a bespoke questionnaire to assess perceived risk may have limited our qualitative data to the second component of risk perception – that is, the severity of the outcome – which is present when drivers assess perceived with hindsight (Borowsky & Oron-Gilad, 2013). Given that the first component – that is, the likelihood of the crash – is mainly available in real-time, perhaps future work should consider implementing a button press response when a hazard has been identified by the driver. In addition, not counterbalancing the experimental conditions may have induced order effects. However, we deemed it necessary to ensure the driving was immersive and realistic. In addition, recalibrating the fNIRS signal for each block was not feasible because it would have extended the length of the simulation and potentially fatigued participants or induced motion sickness.

Although brain activity measures such as fNIRS are not likely to be integrated into production driver monitoring systems (DMS) in the short term – at least not with current wearables – this paper has proven fNIRS to be a helpful research tool for assessing drivers' states. In terms of practical advantages, compared to other common noninvasive measures such as eye-tracking or peripheral physiology, fNIRS can provide a direct measure of complex driver states such as situation awareness (Bracken et al., 2021), trust (Perello-March et al., 2023), driver attention allocation to take-over requests (Fu et al., 2020), or out-of-the-loop states (Balters et al., 2017). fNIRS offers a nearly real-time measure – that is, the haemodynamic response takes only 3–5 seconds – of cortical responses to specific events that can be mapped on the brain regions responsible for certain tasks. For example, fNIRS can be used to measure drivers’ workload during a manual take-over control by mapping prefrontal areas (i.e., known to be responsible for decision-making, working memory, anticipation or judging scenarios) in combination with the motor cortex (responsible for manual tasks or coordinating haptic responses), or measure drivers’ situation awareness levels to visual cues on the road by measuring the activity on the visual cortex (responsible for visual object detection and interpretation). Hence, we encourage future work in this domain to use fNIRS as a complement to gaze-behaviour indicators, behavioural measures, and self-reports or to other physiological indicators to understand better the new challenges arising from automated driving technology.

## CONCLUSION

Overall, this empirical research has evidenced traffic complexity affects risk perception and its derived neurophysiological indicators. Brain oxygenation measures successfully evaluated moderate and high perceived risk levels, indicating drivers actively supervised the vehicle operation in complex traffic scenarios. Our findings evidence the benefits of using fNIRS for driving research to assess driver states and risk in real-time.

## KEY POINTS

- Traffic complexity affects the perception of risk in highly automated driving.
- Increased perception of risks leads to an increase in active monitoring and supervision behaviours with a HAV.
- HAD frees up cognitive resources to evaluate both the likelihood and severity of a possible crash event.
- Females remained more vigilant than males after the driving hazard event.
- fNIRS has proven to be a valuable tool for driver state monitoring.
